# Glutamine relieves oxidative stress through PI3K/Akt signaling pathway in DSS-induced ulcerative colitis mice

**DOI:** 10.22038/ijbms.2020.39815.9436

**Published:** 2020-09

**Authors:** Shuguang Yan, Yi Hui, Jingtao Li, Xiaofan Xu, Qian Li, Hailiang Wei

**Affiliations:** 1 College of Basic Medicine, the Shaanxi University of Chinese Medicine, Xianyang, Shaanxi 712046, P.R. China; 2 Department of Liver Diseases, the Affiliated Hospital of Shaanxi University of Chinese Medicine, Xianyang, Shaanxi 712020, P.R. China; 3 Medical Experiment Center, the Shaanxi University of Chinese Medicine, Xianyang, Shaanxi, 712046, P.R. China; 4 Departments of General Surgery, the Affiliated Hospital of Shaanxi University of Chinese Medicine, Xianyang 712020, Shaanxi, P.R. China

**Keywords:** Colitis, Glutamine, mTOR protein, Oxidative stress, Protein kinase B

## Abstract

**Objective(s):**

Ulcerative colitis (UC) is a kind of complex immune disease, and a major cause of destruction of intestinal barrier and oxidative stress in this field. In this paper, glutamine (Gln) was believed to offer protection against oxidative stress injury in colitis mice.

**Materials and Methods:**

Thirty mice were randomly assigned into control, model, LY294002 (PI3K/Akt inhibitor), Gln, Gln+LY294002 and 5-Aminosalicylic acid (5-ASA) groups. The mice in the experimental group drank 4% dextran sulfate sodium salt (DSS) for 7 consecutive days. The protective effect of Gln on oxidative stress was quantified by keeping colitis mice, involving Phosphatidylinositol-3-kinase (PI3K)/Protein kinase B (Akt)/mammalian target of Rapamycin (mTOR) signaling pathway, with different medications or distilled water through intragastric administration for 10 consecutive days.

**Results:**

*In vivo* administration of Gln, LY294002 or 5-ASA was found to ameliorate the symptoms of colitis in mice, such as reduced growth, loose stools and stool bleeding; protected DSS-induced colitis mice from goblet cell loss, lymphocytosis, mucosal erosion, loss of crypts, and neutrophil infiltration; improved the activity of superoxide dismutase (SOD) and glutathione peroxidase (GSH-XP); decreased the content of malondialdehyde (MDA); and inhibited the activation of PI3K/Akt signaling pathway.

**Conclusion:**

Administration of Gln to the DSS-induced colitis mice led to a clearly reduction in oxidative stress-induced injury. The Gln is confirmed as inhibiting the PI3K/Akt signaling pathway activity.

## Introduction

Ulcerative colitis (UC) is an important subtype of idiopathic inflammatory bowel diseases (IBD) and a major cause of recurrent abdominal pain, blood in stool and diarrhea (1). The people aged 18-50 years are prone to this disease (2). UC is characterized by chronic inflammation and mucosal damage, and its pathogenesis is still unclear. Excessive activation of oxidative stress (OS) is a newly discovered pathological link related to UC in recent years (3). Furthermore, the balance of reactive oxygen species (ROS) generation and antioxidant defense can be impaired by OS (4). The reduction of OS is related with superoxide dismutase (SOD), which is one of antioxidant enzymes. The activity of SOD decreases with increasing of inflammatory process, suggesting that insufficient antioxidant activity is a major cause of inflammation in patients with UC (5). Therefore, the management of anti-oxidative stress plays a key role in UC treatment. 

Glutamine (Gln), a kind of non-essential amino acid with a variety of biological functions, is mainly used to improve various stress responses of the body and to maintain the normal function of immune cells (6). Previous studies have shown that Gln protects multiple organs of the body, especially the intestinal tract, and can alleviate the damage of multiple stress reactions, such as trauma and infection, on the intestinal mucosal barrier function (7-9). Dietary supplementation with Gln had a good effect on intestinal oxidative stress parameters in newborn rats (10). The burned rat model treated with Gln has less intestinal inflammation and oxidative stress due to altered inducible nitric oxide synthase (iNOS) gene aberrant methylation (11). UC patients may benefit from an elemental diet enriched with Gln (12). However, it is not clear that the level of oxidative stress can play protective role in dextran sulfate sodium salt (DSS)-induced colitis mice under treatment with glutamine. 

Phosphatidylinositol-3-kinase (PI3K)/Protein kinase B (Akt) signaling pathway makes important regulatory effects in the occurrence and development of oxidative stress and inflammation (13). UC-associated colon cancer is confirmed to be up-regulated with PI3K/Akt signaling pathway (14). UC patients with inflammation are treated with blocking the PI3K/Akt signaling pathway, which inhibits the activation of nuclear factor kappa B (NF-κB) and reduces the release of cytokines (15). However, relationship between Gln and oxidative stress may be related with the PI3K/Akt signaling pathway, which needs to be studied. 

## Materials and Methods

***Reagents***

Dextran sulfate sodium salt (DSS), LY294002 (PI3K/Akt inhibitor), 5-Aminosalicylic acid (5-ASA) and glutamine (Gln) were all obtained from Sigma-Aldrich (Merck KGaA, Darmstadt, Germany). LY294002 was used at a concentration of 10 µM. Other reagents are of analytical purity in this study.

***Animals***

A total of 30 male Balb/c mice (16-20 g, 6 weeks old) were purchased from the animal center of the West China Medical College of Sichuan University, Chengdu, China. Animal experiments were performed in accordance with the Regulations of Experimental Animal Administration. Mice were housed in polycarbonate cages in an animal laboratory with 12 hr light/12 hr dark cycle under conditions of controlled temperature 24±1˚C and in a humidified atmosphere (50±10%). The animals had* ad libitum* access to food and water for a week, and fasted overnight before inducing colitis and starting the experiment (1, 16). All experiments were performed in accordance with the National Institutes of Health Guide for the Care and Use of Laboratory Animals (NIH Publications No. 8023, revised 1978) and were approved by the Ethical Committee of the west China Hospital of Sichuan University (Chengdu, China).

***Experimental design***

Mice were randomly assigned into six mutually exclusive groups, including the control, LY294002, Gln, Gln+LY294002, model and 5-ASA, with a week of adaptation. Each group contained 5 animals with appropriate treatments. The mice were kept with a compliant diet and water in control group, and were given 4% DSS solution to drink freely for 7 consecutive days in other groups (17, 18). Meanwhile, the mice were given 10 μl/kg/d LY294002 by intraperitoneal (IP) administration in LY294002 group, mice were given 300 mg/kg/d Gln by intragastric administration in Gln group, mice were given 10 μl/kg/d LY294002 by IP administration and 300 mg/kg/d Gln by intragastric administration in Gln+LY294002 group, and the mice were given 50 mg/kg/d 5-ASA by intragastric administration in 5-ASA group. The treatment was provided once a day for 10 consecutive days. The weight loss and disease activity index (DAI) were the major factors to evaluate the UC (19). The weight of each mouse was recorded during the whole experiment. The DAI were assessed daily, including weight loss, stool consistency, and stool bleeding, and scored 0-4 to the severity of each parameter (Table 1). To alleviate the mice pain, the procedure was terminated when the DAI score reached 12 points and the animals were sacrificed by cervical dislocation after being anesthetized by an IP injection of 10% chloral hydrate (350 mg chloral hydrate/kg body weight) for measuring the colon length and spleen weight. 

***Material and specimen handling***

Mice were sacrificed by cervical dislocation after being anesthetized by an IP injection of 10% chloral hydrate (350 mg chloral hydrate/kg body weight) at day 10 following induction with 4% DSS and medical interventions. The entire colon was placed in PBS solution and the length was measured by a ruler. The spleen was taken and weighed, and the distal colon was sliced into three segments of 3, 3, and 2 cm lengths, respectively (21). The 2 cm long segment was fixed in 4% paraformaldehyde solution, and other two segments were preserved at -80 ^°^C.

***Assessment of histological lesions***

Following the colonic tissues was fixed with 4% paraformaldehyde over 24 hr, they were embedded in paraffin blocks to prepare 4-μm-thick paraffin sections. The histological score of samples was assessed by hematoxylin and eosin stain and using an optical microscope (Table 2). H&E-stained colonic tissue sections were scored by a blinded observer using a previously published system (18){Y, 2018 #3292}. 

***Detection of superoxide dismutase, glutathione peroxidase and malondialdehyde contents***

The minced colon and 1:4 (w/v) lysis buffer were homogenized for 30 min, and then the supernatant was collected after centrifuging at 10,000 g for 30 min at 4°C. The colorimetry method, by a microplate reader (Nanjing Jiancheng Bioengineering Institute, Jiangsu, China), was used to detect malondialdehyde (MDA) content, glutathione peroxidase (GSH-Px) activity, and SOD activity. The concentration of MDA in colon, SOD and GSH-Px were expressed in nmol/mg protein, U/mg protein and U/mg protein, respectively.

***Western blot assay***

Protein samples were prepared and quantified from colonic tissues of mice using RIPA lysis buffer (Boster, Wuhan, China) and an Enhanced BCA Protein Assay Kit (Beyotime, Shanghai, China). Following denaturation by boiling, the protein samples (20 μg) were separated on 10% SDS-PAGE gel, and transferred to a PVDF membrane (EMD Millipore, MA, USA). Before incubated with primary antibodies at 4 ^°^C overnight, membranes were blocked with 5% bovine serum albumin at room temperature for 1 hr. The primary antibodies including PI3K (ab154598), p-AKT (ab38449), phospho-mammalian target of Rapamycin (p-mTOR) (ab84400) and β-actin (ab8227) were obtained from Abcam at 1:1,000 dilution. The membrane was washed with TBST followed by incubation with HRP-conjugated goat anti-rabbit IgG secondary antibody (1:5000; ab6721; Abcam, CA, UK) for 1 hr at room temperature. The blots were visualized using an ECL chemiluminescence kit (EMD Millipore). β-actin could act as inner loading control.

***Statistical analysis***

Statistical analysis was performed using SPSS 20.0 (IBM Corp., Armonk, NY, USA). The data are exhibited as the mean±standard deviation. Differences among multiple groups were compared by one-way analysis of variance (ANOVA) with Dunnett’s *post hoc* test or two-way ANOVA with Bonferroni’s *post hoc* test. *P*<0.05 was considered to indicate a statistically significant difference, and <0.01 were considered highly significant.

## Results

***Gln and LY294002 in combination ameliorated DAI score, colon length and spleen index in DSS-induced colitis mice***

**Figure 1:**
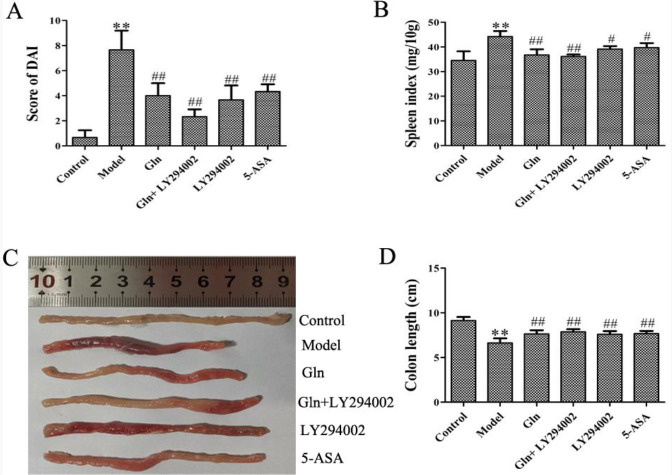
Effects of Gln combined with LY294002 on DAI score, colon length and spleen index in DSS-induced colitis mice

As shown in Figure 1, the DAI scores and spleen index in DSS-induced colitis mice were significantly increased, and colon length in model group decreased significantly comparing with that in control group. Accidie, loose stools and stool bleeding appeared in mice with modeling on the 7th day in the model group. Following medical interventions, the DAI scores and spleen index in DSS-induced colitis mice were significantly decreased, and colon length increased significantly comparing with that in model group. The mice in the treatment group had an obvious improvement, including behavioral activities, regarding feeding and fur appearance. Among them, Gln combined with LY294002 provided the most significant effect.

***Gln and LY294002 in combination ameliorated histopathology in DSS-induced colitis mice***

**Figure 2:**
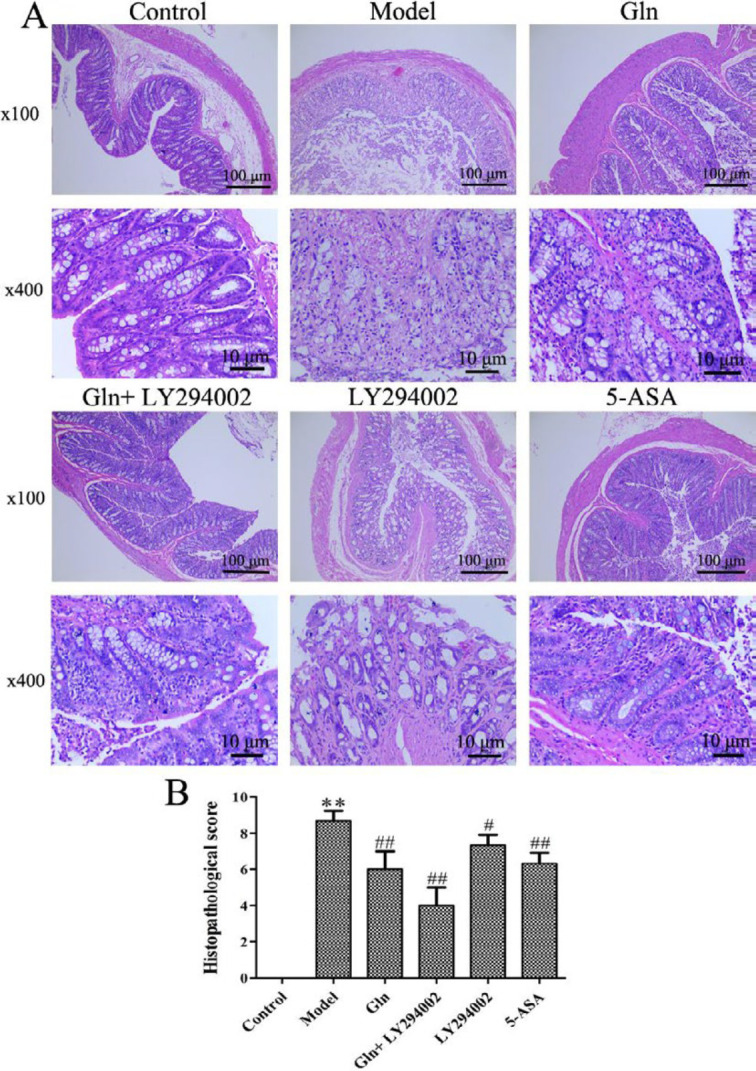
Effects of Gln combined with LY294002 on histological lesions in DSS-induced colitis mice

The histological characteristics of the colon were further assessed by H&E staining. As shown in Figure 2, the mice in control group had no histological injury, whereas mice exposed to DSS in the model, LY294002, Gln, Gln+LY294002 and 5-ASA groups exhibited severe damage of colonic mucosal structure accompanied by disappearance of goblet cells, neutrophils and lymphocytes infiltration. The histological status of LY294002, Gln, Gln+LY294002 and 5-ASA groups was significantly better than that of the model group, among which the Gln+LY294002 group was the best and the histological damage score was the lowest. So, the effect of Gln could be improved by blocking PI3K/Akt signaling pathway on colonic histopathology in DSS-induced colitis mice. 

***Gln strengthens the antioxidant capacity in DSS-induced ulcerative colitis mice through inhibition of the PI3K/Akt signaling pathway in vivo***

**Figure 3:**
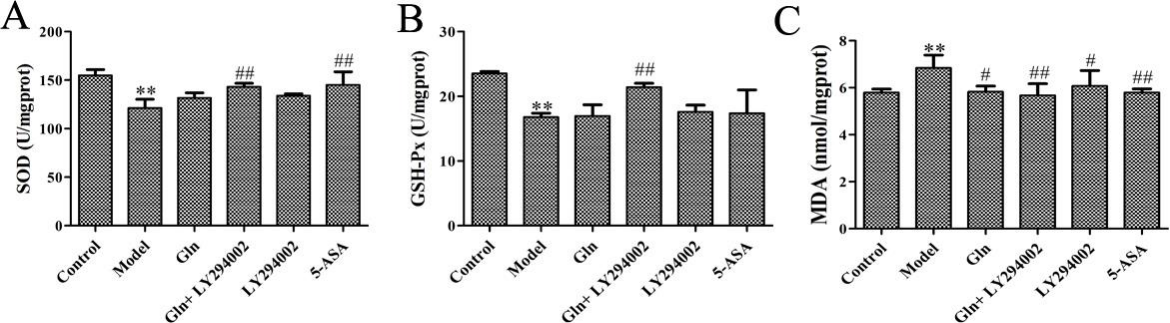
Effect of Gln combined with LY294002 on activity of SOD and GSH-Px and content of MDA in DSS-induced colitis mice

Figure 3 shows that the activity of SOD, GSH-Px and content of MDA had obvious deterioration in model group comparing with control group. The activity of SOD, GSH-Px and content of MDA had obvious improvement in the groups treated with Gln, LY294002 or 5-ASA comparing with the model group. So, the antioxidant capacity of Gln could be improved by blocking PI3K/Akt signaling pathway in DSS-induced colitis mice.

***Gln treatment inhibits the activation of PI3K/Akt signaling pathway in vivo***

**Figure 4:**
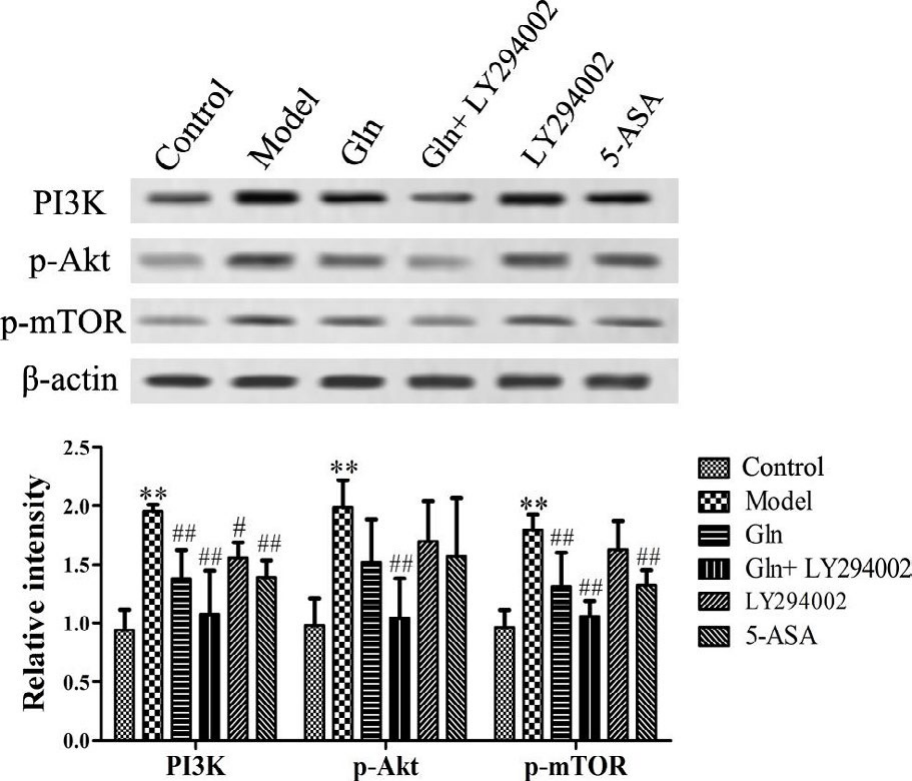
Effect of Gln combined with LY294002 on the expression levels of PI3K, p-AKT and p-mTOR proteins in DSS-induced colitis mice

PI3K/Akt signaling pathway plays an important role in the pathogenesis of colitis (15). In the present study, DSS treatment significantly increased the expression level of PI3K and the Akt and mTOR phosphorylation compared to those noted in the control group. The expression level of PI3K and Akt and mTOR phosphorylation were decreased by varying degrees in LY294002, Gln, Gln+LY294002 and 5-ASA groups compared to those of the model group and markedly increased compared to those of the control group (Figure 4). So, the Gln had a positive inhibitory effect on the activation of the PI3K/Akt signaling pathway. 

**Table 1:** Disease activity index score in DSS-induced colitis mice

Types	Percent weight loss (%)	Stool consistency	Hematochezia level
0	0	Normal	Negative
1	1-5	/	/
2	5-10	Mushy	Positive
3	10-15	/	/
4	>15	Diarrhea	Visible blood in stool

**Table 2:** Histological grading of colitis in DSS-induced colitis mice

Score	Inflammation	Extent	Crypt damage	Percent involvement (%)
0	None	None	None	None
1	Slight	Mucosa	Basal 1/3 damaged	1-25%
2	Moderate	Mucosa and sub-mucosa	Basal 2/3 damaged	26-50%
3	Severe	Transmural	Only surface epithelium intact	51-75%
4			Entire crypt and epithelium lost	76-100%

(A) The DAI scores in each group. (B) The spleen index in each group. (C) Visual appearance of colon. (D) The colon length in each group. The results were presented as the mean±standard deviation. **P<0.01 vs control group; #*P<*0.05 and ##*P<*0.01 vs model group (n=5)

Gln: glutamine; DAI: disease activity index; DSS: dextran sulfate sodium salt; 5-ASA: 5-aminosalicylic acid

(A) Colonic histological lesions in DSS-induced colitis mice (magnification, x100 and x400). (B) The histological score in each group. The results were presented as the mean±standard deviation. **P<0.01 vs control group; ##*P<*0.01 vs model group (n=5)

Gln: glutamine; DAI: disease activity index; DSS: dextran sulfate sodium salt; 5-ASA: 5-aminosalicylic acid

(A) SOD activity in each group. (B) GSH-Px activity in each group. (C) MDA content in each group. The results were presented as the mean±standard deviation. **P<0.01 vs control group; #P<0.05 and ##P<0.01 vs model group (n=5)

Gln: glutamine; DSS: dextran sulfate sodium salt; 5-ASA: 5-aminosalicylic acid; SOD: superoxide dismutase; GSH-Px: glutathione peroxidase; MDA: malondialdehyde

The expression levels of PI3K, p-AKT and p-mTOR protein were examined by western blotting assay. The results were presented as the mean ± standard deviation. **P<0.01 vs control group; #*P<*0.05 and ##P<0.01 vs model group (n=5)

Gln: glutamine; DSS: dextran sulfate sodium salt; 5-ASA: 5-aminosalicylic acid; PI3K: Phosphatidylinositol 3-kinase; p-AKT: phospho- protein kinase B; p-mTOR: phospho-mammalian target of Rapamycin

## Discussion

UC results from oxidative stress and inflammation, which also leads to deterioration of this disease. Currently, the clinical treatment of IBD is mainly using anti-inflammatory drugs such as corticosteroids, aminosalicylate and immunosuppressants, but these drugs have serious side effects (22). Gln, the most abundant free amino acid in plasma, has been shown to play an active role in reducing oxidative stress. It has been revealed that a Gln -enriched elemental diet may be therapeutically beneficial in patients with inflammatory bowel disease (23). Therefore, the present study hypothesized that Gln could reduce the oxidative stress in the intestinal tract of DSS-induced colitis mice, thereby controlling the progression of colitis. 

The animal with trinitrobenzene sulfonic acid (TNBS) and DSS-induced colitis, which are common mechanisms of the pathogenesis of UC, always serves as study model of IBD (24, 25). The similar clinical symptoms and pathological developments display in UC humans and colitis mice, where the mice is treated with DSS. DSS has a direct toxic effect on the intestinal epithelium, which can lead to the erosion of the intestinal epithelium, eventually destroying completeness of the mucosal barrier (26). So, the colitis model was established by injecting DSS solution into normal mice. The improvement in the physical barrier with Gln is studied to evaluate the effects in this research. In this study, DSS can make colon shortening, weight loss and DAI, which are the routine clinical indicators. Following the administration of different medications, the symptoms of colitis in mice were relieved, and the Gln shows a similar therapeutic effect with 5-ASA, a positive therapeutic agent for colitis treatment. Inflammatory response is related with histopathologic damage (27); it has been revealed that Gln combined with LY294002 has protected the mice against mucosal erosion, goblet cell loss, neutrophil infiltration, lymphocytosis, and loss of crypts caused by DSS-induced colitis. These results suggested that the inhibition of PI3K/Akt signaling pathway can enhance the protective effect of Gln on DSS-induced colitis in mice. 

Gln is a non-essential amino acid, but it acts a pivotal part in the survival and growth of intestinal cells. The stress can be effectively relieved with Gln, which leads to evident reduction of inflammation and oxidative stress for treating intestinal diseases (11, 28). Oxidative stress can activate macrophages and neutrophils due to pro-inflammatory factors entering the colon, which produces superoxide and ROS (29). The present study revealed that the Gln combined with LY294002 restored DSS-induced colitis mice with reducing GSH-Px activity, SOD, and increasing MDA content, which cause the enhancement of antioxidant capacity in Gln with inhibiting PI3K/Akt signaling pathway. PI3K is an intracellular phosphatidylinositol kinase, which produces the second messengers of inositol lipid substances (30). PI3K has a direct downstream target, which is called Akt (31). Huang *et al.* (15) reported that the PI3K/Akt signaling pathway has a positive correlation with UC through regulating and releasing pro-inflammatory cytokines such as tumor necrosis factor -alpha (TNF-a). Worou *et al.* (32) reported that Hemin reduces cardiac oxidative stress in systemic hypertension mice through the PI3K/Akt signaling pathway. Therefore, the present study speculated that Gln could treat mice with UC by anti-oxidative stress with the PI3K/Akt signaling pathway. 

## Conclusion

The findings of present study suggest that Gln could protect against oxidative stress-induced injury in colitis mice through inhibiting the PI3K/Akt signaling pathway. This study may provide potential therapeutic strategies for the future treatment of colitis and a possible effect for Gln to improve colitis. Considering the complexity of the pathogenesis of UC, further efforts are needed to confirm this finding.
